# Transcriptional activities of human elongation factor-1α and cytomegalovirus promoter in transgenic dogs generated by somatic cell nuclear transfer

**DOI:** 10.1371/journal.pone.0233784

**Published:** 2020-06-03

**Authors:** Kiyoung Eun, Nayoung Hong, Yeon Woo Jeong, Min Gi Park, Seon-Ung Hwang, Yeon I. K. Jeong, Eun Ji Choi, P. Olof Olsson, Woo Suk Hwang, Sang-Hwan Hyun, Hyunggee Kim

**Affiliations:** 1 Department of Biotechnology, College of Life Sciences and Biotechnology, Korea University, Seongbuk-gu, Seoul, Republic of Korea; 2 Institute of Animal Molecular Biotechnology, Korea University, Seongbuk-gu, Seoul, Republic of Korea; 3 Sooam Biotech Research Foundation, Guro-gu, Seoul, Republic of Korea; 4 Laboratory of Veterinary Embryology and Biotechnology, College of Veterinary Medicine, Chungbuk National University, Seowon-gu, Cheongju, Republic of Korea; 5 Institute of Stem Cell & Regenerative Medicine, Chungbuk National University, Seowon-gu, Cheongju, Republic of Korea; Justus Liebig Universitat Giessen, GERMANY

## Abstract

Recent advances in somatic cell nuclear transfer (SCNT) in canines facilitate the production of canine transgenic models. Owing to the importance of stable and strong promoter activity in transgenic animals, we tested human elongation factor 1α (hEF1α) and cytomegalovirus (CMV) promoter sequences in SCNT transgenic dogs. After transfection, transgenic donor fibroblasts with the hEF1α-enhanced green fluorescence protein (EGFP) transgene were successfully isolated using fluorescence-activated cell sorting (FACS). We obtained four puppies, after SCNT, and identified three puppies as being transgenic using PCR analysis. Unexpectedly, EGFP regulated by hEF1α promoter was not observed at the organismal and cellular levels in these transgenic dogs. EGFP expression was rescued by the inhibition of DNA methyltransferases, implying that the hEF1α promoter is silenced by DNA methylation. Next, donor cells with CMV-EGFP transgene were successfully established and SCNT was performed. Three puppies of six born puppies were confirmed to be transgenic. Unlike hEF1α-regulated EGFP, CMV-regulated EGFP was strongly detectable at both the organismal and cellular levels in all transgenic dogs, even after 19 months. In conclusion, our study suggests that the CMV promoter is more suitable, than the hEF1α promoter, for stable transgene expression in SCNT-derived transgenic canine model.

## Introduction

Transgenic animal models are widely used in both basic research and preclinical studies. In particular, transgenic animal models of various human diseases provide vital information regarding biological phenomena occurring in humans [1]. Over the past two decades, genetic engineering techniques such as zinc-finger nucleases-, Cre/loxP-, and CRISPR/Cas9-based site-specific genome editing methods, and gene silencing by RNA interference, have been developed and optimized for various human and animal systems. These technologies have resulted in a better platform for producing transgenic animals [2–4]. In addition, several techniques have been developed, which assist in transgenic animal production, including pronuclear microinjection and somatic cell nuclear transfer (SCNT) [5]. These techniques facilitate the generation of transgenic animals with the exogenous genetic materials necessary for the intended model system. Recently, SCNT has been utilized to produce transgenic models of large animals even those with a relatively long generation interval [6]. By combining genetic engineering systems and animal reproductive technologies, the potential of generating alternative disease models, with physiological and genetic characteristics, that recapitulate those in humans, has become more feasible [7, 8].

Dogs, especially pet dogs, are one of the most important animals in veterinary science due to the presence of extensive clinical database regarding various diseases including cancers [9]. In addition to its importance in veterinary research, these databases are applicable, particularly in preclinical studies, as canine diseases are highly similar to human diseases with respect to pathologic and genetic characteristics [10, 11]. In oncology and other disease-based research, the use of transgenic as well as spontaneous disease models provides a more comprehensive understanding of medical and veterinary science [12]. However, due to the many hurdles in canine cloning using SCNT, only a few reports on the generation of transgenic dogs have been published. This current situation causes limitations in the selection of optimal genetic engineering elements for the development of effective transgenic canine models due to a lack of referenceable precedent studies.

Many studies have reported that transgenes are frequently and permanently silenced after transfection or generation of transgenic animals [13–15]. Therefore, it is important to design a gene expression vector system capable of inducing the expression of the transgene and to demonstrate a clear *in vivo* phenotypic expression. This requires the use of appropriate genetic elements, including promoters, introns, protein coding sequences, and polyadenylation signals [16]. First of all, the most crucial point is to determine which promoter sequence is optimal for driving ectopic expressions in cellular and animal models, as promoters are the most direct regulators of transgene expression [17–19]. In this study, we compared, the most widely used promoter sequence for considerably strong and stable transgene expression, human elongation factor 1α (hEF1α) and Cytomegalovirus (CMV) promoter, in SCNT transgenic dogs by detecting enhanced green fluorescence protein (EGFP).

## Materials and methods

### Ethics statement

All animal procedures were conducted in accordance with the animal study guidelines and approved by the Sooam Ethical Committee for Animal Experiment of the Sooam Biotech Research Foundation, Korea (permit no. C-16-01). Female mixed breed dogs (Hyundae Kennel, Seoul, Korea), aged 1 to 7 years (body weight 20–25kg), were housed in indoor kennels (2.5 × 1.5 m) under a 12hr/12hr light/dark cycle with natural light; these dogs were fed standard commercial dog food once a day, and given water *ad libitum*. The indoor temperature was set at 22°C (range 18–24°C) and the humidity was 50% (range 40–70%) with 10–15 fresh air exchanges per hour. All dogs received 40 min of supervised group exercise and socialization daily in a separate fenced yard (28.3 × 16 m). All dogs used in this study were returned to their colony for retirement or adoption. All surgical procedures were performed under general anesthesia by veterinarians. Humane endpoint criteria and euthanasia, to minimize pain or suffering—in accordance to the guidelines proposed by the American Veterinary Medical Association—were based on observation of chronic pain or distress, which could not be mitigated or controlled with medication. When the determined endpoint criteria were met, the animals were euthanized without delay. Two newborn pups, BTM-876 and BTM-882 did not manifest noticeable pain or distress, and therefore not euthanized, but succumbed to death right after birth. BTF-967 was euthanized under anesthesia with tiletamin/zolazepam (Zoletil, Virbac, France) followed by an injection of 20 ml KCl (150 mg/ml) after 54 days birth due to pain from a brain cancer and a sarcomatoid carcinoma in the left shoulder muscle. Unless the rest of newborn pups, BTM-881, BTM-884, BTF-963, BTF-964, BTF-965, BTF-966, and BTF-968, have any problem, they will be maintained under the appropriate care until the end of their lives.

### Cell culture and transfection

The dog fetal fibroblast cells were maintained in complete cell culture medium, Dulbecco’s modified Eagle’s medium (DMEM; Life Technologies, CA, USA), supplemented with 10% (v/v) fetal bovine serum (FBS), 1% (v/v) Glutamax (Life Technologies), 1% (v/v) non-essential amino acids (Life Technologies), 1% (v/v) antibiotic-antimycotics (Life Technologies), and 0.1% (v/v) 2-mercaptoethanol (Life Technologies). The incubation conditions for the primary culture were 37°C and 5% CO_2_ in a humid incubator.

Transfection of each linearized plasmid vector into canine fibroblasts was performed using LipoJet^™^ In Vitro Transfection Kit (SignaGen Laboratories, MD, USA) according to the manufacturer’s instructions. EGFP expression after transfection was monitored by IncuCyte ZOOM^™^ (Essen BioScience, MI, USA) and average green object intensity per image was calculated using IncuCyte ZOOM^™^ software.

### Primary fibroblast isolation

Fibroblasts were isolated from dog (*Canis lupus familiaris*) fetuses on day 39 of gestation and from the ear skin of each pup as previously described [20]. Briefly, tissues from fetus were washed in phosphate-buffered saline (PBS), containing 1% (v/v) antibiotic-antimycotic (Life Technologies) and chopped finely using a blade. Chopped skin tissues were incubated with 0.25% trypsin for 2 h and then added to the complete cell culture medium to neutralize the trypsin. Trypsinized tissues were cultured in a 100-mm culture dish and adherent cells were subcultured. The incubation conditions for the primary culture were 37°C and 5% CO_2_ in a humid incubator.

### Vector construction

To construct hEF1α-EGFP and CMV-EGFP plasmid constructs, LCMV:ECFP(loxP)(FRT)EYFP (#31304; Addgene, MA, USA) was modified by gene cloning. EYFP sequence was eliminated using *Sma1* and *Kpn1*. The ECFP gene was replaced with the EGFP sequence using *NcoI* and *BsrGI* from EGFP-C2 (#6083–1; Clontech Laboratories Inc., CA, USA). The following genes and elements were obtained by PCR amplification: Human EF1α promoter (from pCDH-CMV-MCS-EF1-puro; System Biosciences Inc., CA, USA), CMV promoter (from pcDNA^™^-3.1^(+)^; Thermo Fisher Scientific, MA, USA). PCR amplification process was performed using Extaq^®^ DNA polymerase (Takara Bio Inc., CA, USA) and the following primers: hEF1α, forward (F) 5′-aggactctcactttctctctctgc-3′ and reverse (R) 5′-tgcaggctttatggaggagt-3′; CMV, (F) 5′-gggccagatatactcgttga-3′ and (R) 5′-gccagagagctctgcttat-3′. After amplification, each product was ligated to pGEM^®^-T Easy Vector (Promega, WI, USA) according to the manufacturer’s instructions and verified by sequencing analysis (BIONICS Corp., Seoul, Korea). Finally, they were digested with each of the designated restriction enzymes and ligated into the plasmid vector.

### Donor cell line construction and fluorescence activated cell sorting (FACS)

Canine fibroblasts were transfected with linearized hEF1α-EGFP and CMV-EGFP plasmid vector using an electroporation tool, Neon^®^ Transfection System (Invitrogen, CA, USA), according to the manufacturer’s instructions. To isolate the GFP-positive cells, canine cells attached on a culture plate were dissociated into single cells by trypsinization and washed twice with PBS. Then, GFP-positive cells were sorted by FACS Aria II (BD biosciences, CA, USA). GFP expression in primary fibroblasts derived from transgenic pups was analyzed by FACS verse (BD Biosciences).

### Genomic DNA isolation and PCR analysis

To identify transgenic puppies, genomic DNA of all of puppies was analyzed. Each genomic DNA sample was isolated from the fibroblast cells of all born puppies using Wizard^®^ Genomic DNA Purification Kit (Promega, WI, USA) according to the manufacturer's instructions. Transgenes in the genomic DNA were amplified with each primer set and NeoTherm^™^ DNA polymerase (Genecraft, Manchester, UK) or PrimeSTAR^®^ GXL DNA Polymerase (Takara Bio Inc.). The primers and primer combinations used for PCR amplification are as follows. The primers are hEF1α-EGFP (F), 5′-aaggatctgcgatcgctccg-3′ and (R), 5′-ttttcgcaacgggtttgcc-3′; CMV-EGFP (F) 5′-gcctcttcgctattacgcca-3′ and (R) 5′-taggtcagggtggtcacgag-3′; EGFP (F), 5′-cctgaagttcatctgcacca-3′ and (R), 5′-cttgtacagctcgtccatgc-3′; GAPDH (F), 5′-ggtagtgaagcaggcatcgg-3′ and (R), 5′-ttactccttggaggccatgtg-3′.

For inverse PCR to identify CMV-EGFP transgene integration site in transgenic dogs, 2 μg genomic DNA from each BTF-965 and BTF-968 dogs was digested with *BamH1* (Takara Bio Inc.) and *Xma1* (Takara Bio Inc.) at 37 °C overnight. Then, restriction enzymes were inactivated at 75 °C for 20 min. 50 ng of digested DNA was self-ligated using T4 ligase (Enzynomics, Inc., Daejeon, Korea) at 4 °C overnight. The first-round of PCR was performed using forward primer, 5′-GCCAAGTACGCCCCCTATTG-3′, and reverse primer, 5′-ATGGAAAGTCCCTATTGGCGT-3′, and PrimeSTAR^®^ GXL DNA Polymerase using the following thermal cycling conditions: 60 s at 98 °C; 35 cycles of 15 s at 98 °C, 15 s at 58 °C, and 450 s at 72 °C; followed by cooling at 4 °C. Then, the second-round PCR was performed using forward primer, 5′- GGGACTTTCCTACTTGGCAGT-3′, and reverse primer, 5′- GGCTATGAACTAATGACCCCGTA-3′, under PCR conditions identical to those used in first-round PCR. PCR products were separated by DNA electrophoresis on a 1% agarose gel, and in-gel DNA extraction was performed using a gel extraction kit (Elpis Biotech Inc., Daejeon, Korea) according to the manufacturer’s instructions. Finally, the PCR product was sequenced using an ABI BigDye^®^ terminator v3.1 Cycle Sequencing kit (Applied Biosystems, CA, USA) at Bionics Corp. (Seoul, Korea).

### Immunohistochemistry

Brain and muscle tissue samples of BTF-967 dog, euthanized because of a cancer development, were used to verify *in vivo* EGFP expression in transgenic dogs. Tissues were fixed with 4% paraformaldehyde (Sigma-Aldrich, MO, USA) and embedded in paraffin, sectioned (4μm in thickness), and placed on glass slides. After deparaffinization and hydration, tissue sections were microwaved for 20 min in sodium citrate buffer (pH 6.0) for antigen retrieval using the buffer containing 10 mM sodium citrate (SAMCHUN Chemical, Seoul, Korea) and 0.05% Tween-20 (LPS Solution, Daejeon, Korea). Endogenous peroxidase was blocked in a 9:1 mixture of methanol (DUKSAN Science, Seoul, Korea) and 30% H_2_O_2_ (Sigma-Aldrich) for 10 min. Then, tissue slides were stained with primary antibodies against EGFP (1:1,000; ab290, Abcam, Cambridge, UK) and RFP (1:200; 600-401-379s, Rockland Immunochemicals Inc., PA, USA), for 12 h at 4 °C. Then, tissues were washed twice with PBS and incubated with biotinylated secondary antibody (Vector Laboratories, Inc., CA, USA) for 1 h at room temperature. Finally, all samples were developed using the Vectastain ABC horse radish peroxidase (HRP) (Vector Laboratories, Inc.) and DAB peroxidase substrate kits (Vector Laboratories, Inc.) according to the manufacturer’s instructions. The tissue sections were subsequently counterstained using hematoxylin.

### Quantitative reverse transcription-polymerase chain reaction (qRT-PCR) and semi-quantitative PCR analysis

All RNA samples were isolated from each cells using TRIzol Reagent (Invitrogen) according to manufacturer’s instructions. RNA (1 μg) that had been treated with RNase-free DNase was utilized as a template for synthesizing complementary DNA (cDNA) using the RevertAid First Strand cDNA Synthesis Kit (Thermo Fisher Scientific,) according to manufacturer instructions. qRT-PCR analysis was performed using Takara Bio SYBR Premix Ex Taq and CFX096 (Bio-Rad, CA, USA). The expression level of the EGFP gene was normalized to that of GAPDH. The primer sequences used in this experiment are as follows: dog GAPDH (F), 5′-ggtagtgaagcaggcatcgg-3′ and (R), 5′-ttactccttggaggccatgtg-3′; EGFP (F), 5′-cctgaagttcatctgcacca-3′ and (R), 5′-aagtcgtgctgcttcatgtg-3′.

In case of BTM-876, the expression level of EGFP gene was detected by semi-quantitative PCR, due to its undetectable expression in the DMSO treated group. PCR was performed using the first pair of primers and PrimeSTAR^®^ GXL DNA polymerase (Takara Bio Inc.) with the following thermal cycling conditions: 30 cycles of 10 s at 98°C, 15 s at 60°C, and 70 s at 68°C. EGFP (F), The following primers were used: 5′-cctgaagttcatctgcacca-3′ and (R), 5′-cttgtacagctcgtccatgc-3′.

### Ovulation determination

Unless otherwise indicated, all reagents were obtained from Sigma-Aldrich. All donors and recipients employed in the study showed spontaneous estrus. The estrus stage was examined weekly by observing for vulval bleeding to detect the onset of the heat period. During heat, a 2 mL blood sample was collected daily by cephalic venipuncture and serum P4 levels in the blood samples were measured by electrochemiluminescence immunoassay (Cobas e411, Roche Diagnostics, Mannheim, Germany; intra- and inter-assay coefficients of variation < 4%). Ovarian ultra-sonographies were periodically performed twice per day when serum P4 levels were found to be increased by more than 2 ng/mL. The time of ovulation was designated as the time when the ovaries became difficult to find due to an apparent decrease in the number or contour of anechoic follicles, or their disappearance anechogenicity by transabdominal ultrasonography and as the proportion of superficial epithelial cells was greater than or equal to 90% of epithelial cells from vaginal swabs, which were stained using Diff Quik (Sysmex Co., Kobe, Japan) based on standard protocols [21].

### Oocyte collection

All oocyte donors and surrogates underwent spontaneous estrus, and donors and surrogates were matched based on the synchronization of their estrus. Oocytes were surgically retrieved at 3–4 days post-ovulation. Before surgery, blood sample was drawn through the cephalic venipuncture, and blood plasma was collected and frozen (-20 °C) for hormone analyses. Anesthesia was induced with a mixture of xylazine hydrochloride (Rumpun^®^; Bayer Korea, Ansan, Korea; 1mg/kg body weight) and ketamine HCl (Ketalar^®^; Yuhan Corp.; 50 mg/mL, Seoul, Korea; 4mg/kg body weight) and maintained with inhalation of isoflurane. Under aseptic conditions, the reproductive tract was exposed through a midventral incision, with care taken to minimize exposure and manipulation of the organs. Corpora lutea (CL) were counted and oocytes were bilaterally flushed, using a catheter, from each oviduct with 10mL TCM 199 supplemented with HEPES (Invitrogen). Oocytes were collected using a stereomicroscope, transferred into fresh medium, and subjected to nuclear transfer.

### Evaluation of retrieved oocytes

The maturation stage of the retrieved oocytes was determined as previously described [22]. The oocytes were stripped of cumulus cells and pre-stained with 5mg/mL Bisbenzimide (Hoechst 33342) to visualize the presence of nuclei for enucleation process. Oocytes were graded based on their morphology and nuclear stage as immature (cumulus very closely attached to oocytes, nuclear stage is either germinal vesicle (GV), GV breakdown, or metaphase I), mature (metaphase II oocytes with several layers of cumulus cells and homogeneous cytoplasm), aged (unidentified nuclear status with the cytoplasmic membrane shrink, metaphase II oocytes in less than 70% of the cytoplasm and loosely attached cumulus cells), abnormal (irregular cytoplasmic contour, protrusion of zona pellucida, nuclear immaturity), or ruptured (oocytes with broken zona and cytoplasmic membrane) under an inverted microscope equipped with epifluorescence (TE2000-E; Nikon Corp., Tokyo, Japan).

### Nuclear transfer

After evaluation of the maturation status, metaphase II oocytes were enucleated by squeezing out the first polar body and metaphase II plate into a small amount of surrounding cytoplasm using a glass pipette. Donor cells were prepared and treated using a conventional system of primary cell culture as described previously [23]. Using a fine pipette, a trypsinized cell with a smooth cell surface was transferred into the perivitelline space of an enucleated oocyte. The couplets were equilibrated with 0.26 M mannitol solution containing 0.5 mM of HEPES, 0.1 mM of CaCl_2_, and MgSO_4_ for 4 min. Next, the couplets were transferred to a chamber with two electrodes and covered with mannitol solution. The couplets were fused with two DC pulses of 1.75–1.85 kV/cm for 15 μs using a BTX Electro-Cell Manipulator 2001 (BTX, Inc., CA, USA). After simultaneous fusion and activation, a group of 5–6 embryos were cultured in 25 μL microdrops of mSOF covered with mineral oil for 1 h at 39 °C in a humidified atmosphere (5% O_2_, 5% CO_2_, and 90% N_2_) until embryo transfer.

### Embryo transfer and pregnancy diagnosis

Surrogate dogs with estrus matching that of oocyte donors were anaesthetized as described previously using an oocyte retrieval procedure. The ovary with a greater number of corpora lutea was approached by ventral laparotomy. The fat layer covering the ovary was gently grasped with forceps and suspended with a suture to exteriorize the fimbriated end of the oviduct. Immediately after fusion and activation, all reconstructed embryos were loaded into a tomcat catheter (3.5 Fr × 5.5”; Sherwood Medical, MO, USA) with at least a medium volume (2–4 μL) and gently transferred into the 2/3 distal position of the oviduct through the infundibulum. Pregnancy was confirmed by transabdominal ultrasound with a real-time ultrasonography at 25–30 days after embryo transfer. Ultrasonography was performed either in the standing or dorsal recumbency position using a portable ultrasound machine with a 3.5 MHz curved transducer (Sonace R7; Samsung Medison, Seoul, Korea). Ultrasonographies were repeated every 7 days on pregnant surrogates until term. The sizes and shapes of the chorionic cavities and presence of an embryonic or fetal heartbeat were examined to identify embryonic or fetal death.

### Statistical analysis

All experiments were replicated more than three times. All data were analyzed by one-way ANOVA (analysis of variance) followed by Duncan’s test using SPSS software (SPSS, Inc., IL, USA) and are reported as the mean ± standard error of the mean. Statistically significant differences were considered significant if the P-value was less than 0.05.

## Results

### Production of SCNT transgenic dogs of EGFP gene controlled by hEF1α and CMV promoter

**Fig 1:**
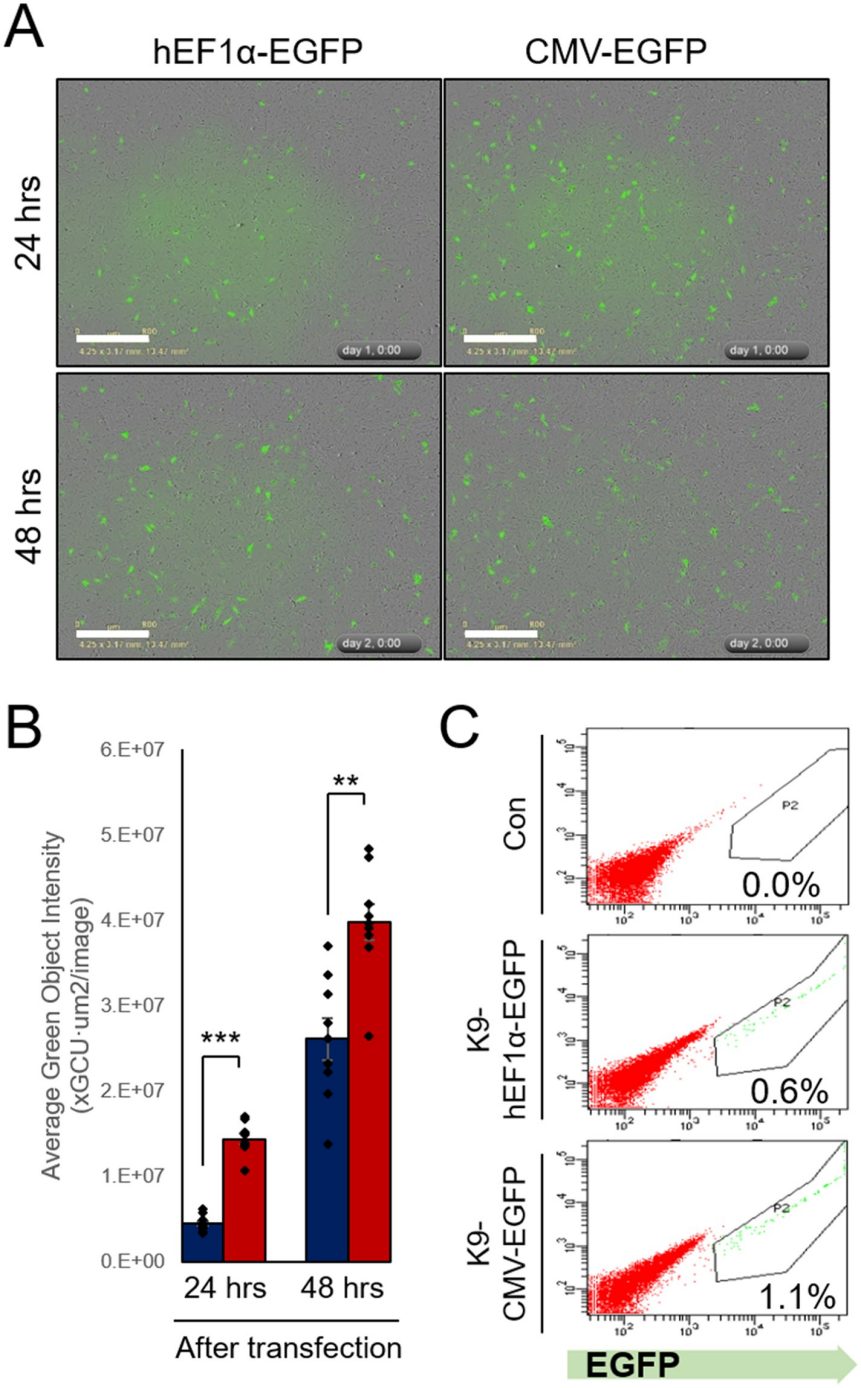
Construction of transgenic canine donor fibroblast with EGFP gene controlled by hEF1α and CMV promoter. (A) Representative images of EGFP-expressing cells at indicated time after transfection with linearized hEF1α- and CMV-EGFP plasmid vectors captured by IncuCyte^®^ equipment. (B) Quantitative data of green fluorescence object segmentation analysis by the IncuCyte^®^ basic software. (C) EGFP-positive transgenic donor cells sorted using FACS, 2 weeks after transfection into canine fetal fibroblasts. Scale bars indicate 800 μm. **, *P* < 0.01. ***, *P* < 0.001.

To compare transcriptional activities of hEF1α and CMV promoter sequences in a transgenic canine model, we used reporter plasmid vectors using an enhanced green fluorescence protein (EGFP) transgene, under the control of the respective promoters. We transfected hEF1α-EGFP and CMV-EGFP vectors into canine fibroblasts and compared EGFP expression levels using IncuCyte^®^ live imaging system during a period of transient expression. Results indicated that the CMV promoter provided a stronger expression level of the transgene than did the hEF1α promoter (Fig 1A and 1B). After 2 weeks, 0.6% and 1.9% of cells expressing hEF1α-EGFP and CMV-EGFP, respectively, were isolated using FACS (Fig 1C). Each cell line was considered to be stably transfected and were named K9-hEF1α-EGFP and K9-CMV-EGFP, respectively.

SCNT using K9-hEF1α-EGFP and K9-CMV-EGFP resulted in an oocyte fusion rate of 67.5% (255 oocytes out of 378) and 49.5% (453 oocytes out of 915), respectively. SCNT embryos were transplanted into 17 and 28 surrogates and three and four respective surrogates were diagnosed with pregnancy (Table 1). Four cloned transgenic puppies originating from hEF1α-EGFP transgenic cell lines were produced. These four pups were named BTM-876, -881, -882, and -884 (Table 2). Unfortunately, BTM-876 was born with hypermyotrophy and macroglossia and died shortly after birth; BTM-882 was also born with hypermyotrophy and died 3 days after birth (Table 2). These deformities are reported to be occasionally found in cloned SCNT puppies produced by transgenic and non-transgenic SCNT. On the other hand, healthy puppies, BTM-881 and BTM-884, did not show any deformities or disabilities 9 months after birth. In case of CMV-EGFP, six dogs, BTF-963, -964, -965, -966, -967, and -968, were born (Table 2). BTF-967 was euthanized 54 days after birth due to spontaneous brain cancer and a sarcomatoid carcinoma in the left shoulder muscle (Unpublished data).

**Table 1:** Pregnancy rate and cloning efficiency of hEF1α-EGFP and CMV-EGFP.

	Nuclear transfer				Embryo transfer			Parturition		
Donor cell	No. of oocyte donor dog	No. of oocyte			No. of Surrogate	No. of pregnancy		No. of total born (%)	No. of offspring (%)	
		Retrieved	Subjected to NT	Fused & Transferred (%)		At mid-term (%)	To term (%)		Abnormal	Live until weaning
K9-hEF1α-EGFP	40	391	378	255 (67.5)	17	3 (17.6)	3 (17.6)	4 (1.6)	2 (0.8)	2 (0.8)
K9-CMV-EGFP	96	1061	915	453 (59.3)	28	4 (14.3)	4 (14.3)	6 (1.3)	1 (0.2)	5 (1.1)

No. of transferred embryos was counted from surrogates carrying a full-term pregnancy.

Percentage was based on the number of transferred embryo.

**Table 2:** Pregnancy results of puppies derived from each cell line.

Donor cell	Pregnant recipient	Cloned offspring	Delivery type	Gestation term (days)	Morphological abnormality	Birth weight (g)	Age at death (days)
K9-hEF1α-EGFP	AS1302	BTM-876	C/S	63	Hypermyotrophy, Macroglossia	360	0
	AS1393	BTM-881		57	-	285	-
		BTM-882			Hypermyotrophy	325	3
	AS1410	BTM-884		60	-	395	-
K9-CMV-EGFP	HD5781	BTF-963	C/S	59	-	285	-
		BTF-964			-	250	-
	AS2407	BTF-965		62	-	375	-
	HD5784	BTF-966		61	-	410	-
	AS2378	BTF-967		59	-	385	54
		BTF-968			-	385	-

C/S: Caesarean section.

### Silencing of hEF1α promoter activity in transgenic canine model by epigenetic modification

**Fig 2:**
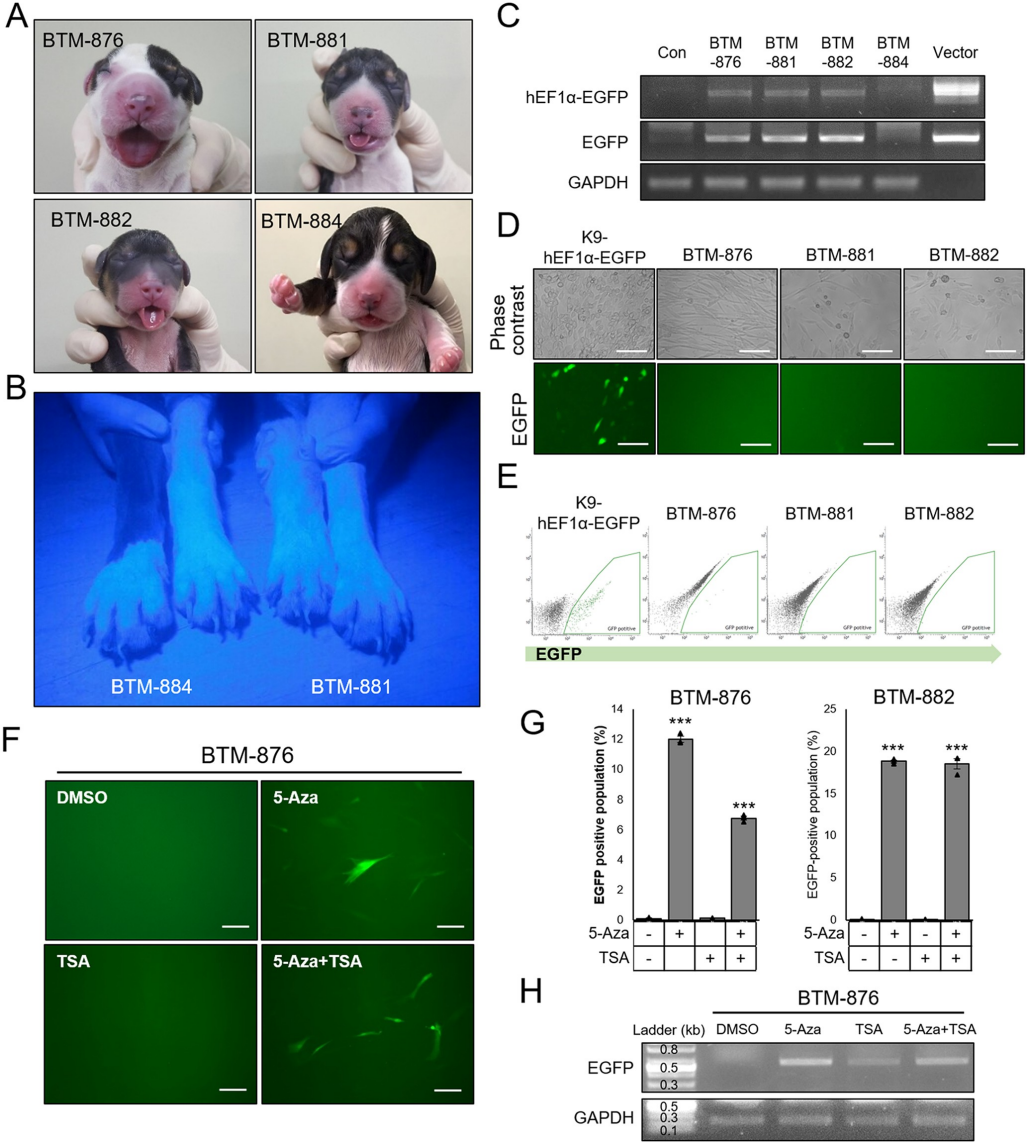
Analysis of transgenic dogs with EGFP controlled by hEF1α promoter sequence. (A) Representative images showing the four pups produced by SCNT using K9-hEF1α-EGFP fibroblasts. (B) Representative image showing living BTM-881 (right) and BTM-884 (left) dogs without detectable EGFP under ultraviolet light exposure. (C) PCR analysis to detect whole hEF1-EGFP or EGFP construct in genomic DNA of each born dog. GAPDH is the loading control. (D) Detection of EGFP signal in fibroblasts isolated from each transgenic dog using fluorescence images and (E) FACS analysis. (F) Representative images of EGFP-expressing cells treated with 10 μM 5-Aza and/or 0.2 μM TSA for 72 hrs. (G) Quantitative date of cytometry analysis showing the percentage of EGFP-expressing cells in each cell after 5-Aza or/and TSA treatment. (H) Semi-quantitative PCR analysis using isolated mRNA from cells in (F). All scale bars indicate 50 μm. ***, *P* < 0.001.

To determine whether the newly born puppies were transgenic, EGFP expression was examined in their claws under ultraviolet light. Unexpectedly, we found that none of the pups expressed green fluorescence, which is easily detectable even at low ultraviolet light intensity (Fig 2A and 2B). Next, we isolated fibroblasts from each cloned pup, to confirm GFP expression at the cellular level. After genomic DNA was extracted from these cells, the presence of transgene in the genome was confirmed by PCR analysis. Three puppies, BTM-876, -881, and -882, had a transgene construct, whereas no transgene was detectable in BTM-884 (Fig 2C, S1 Appendix). We examined EGFP expression in fibroblasts isolated from each of the cloned puppies using FACS and fluorescence microscopy, and found no EGFP expression in pups derived from K9-hEF1-EGFP, although donor cells exhibited strong and stable EGFP expression before conducting SCNT (Fig 2D and 2E). Previous study indicated that treatment with a DNA methyltransferase inhibitor, 5-Azacytidine (5-Aza), and a histone deacetylase inhibitor, Trichostatin A (TSA), reactivated epigenetically silenced transgene expression in transgenic pig fibroblast [24]. We tested whether 5-Aza and TSA treatment could rescue EGFP expression. Some fraction of the 5-Aza or 5-Aza and TSA co-treated cells expressed detectable EGFP, as measured by fluorescence microscopy and FACS (Fig 2F and 2G). Samples of mRNA indicated that 5-Aza strongly reactivated the transcription of the EGFP gene, whereas TSA alone only moderately reactivated the transcription (Fig 2H, S2 Appendix). Collectively, these data indicated that *in vivo* silencing of the EGFP transgene, regulated by the hEF1α promoter sequence, was predominantly due to DNA methylation. This was evident in all of the transgenic canine models generated by SCNT.

### Strong transcriptional activity from the CMV promoter in transgenic canine model

**Fig 3:**
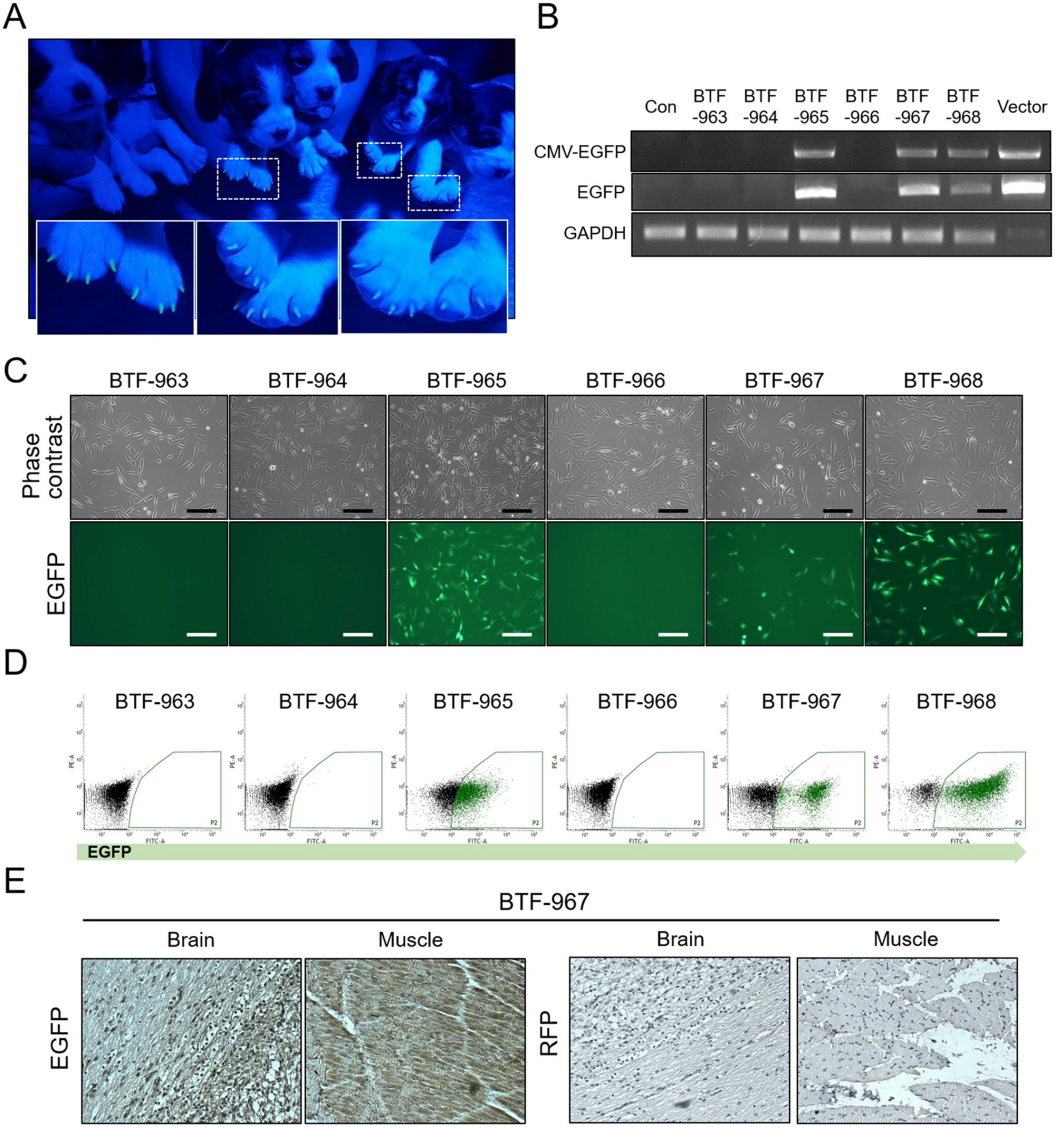
Analysis of transgenic dogs with EGFP controlled by CMV promoter sequence. (A) Representative image of all born puppies under ultraviolet light exposure, to assess EGFP expression, BTF-963, - 964, -965, -966, -967, and -968, from left to right in order. Claws of three puppies, BTF-965,—967, and -968, showed a detectable EGFP signal, but the signal was undetectable in the other three pupies. (B) PCR analysis to detect whole CMV-EGFP or EGFP construct in genomic DNA of each born dog. GAPDH is the loading control. (C) Detection of GFP signals in fibroblasts isolated from each transgenic dog using fluorescence microscopy and (D) FACS analysis. Scale bars indicate 50 μm. (E) Immunohistochemical analysis showing EGFP expression in brain and muscle tissues from BTF-967 dog. RFP is a negative control of the immunohistochemical reaction.

Six cloned dogs produced by SCNT using K9-CMV-EGFP donor cells were analyzed by the same process. The EGFP transgene was detectable in three of the six clones by visualization of their claws under ultraviolet light. We detected EGFP expression in BTF-965, -967, and 968, but not in the remaining three puppies (Fig 3A). PCR analysis confirmed our visual results (Fig 3B, S3 Appendix). BTF-963, -964, and -966 were not found to contain the transgene (Fig 3B). EGFP expression in fibroblasts isolated from cloned pups was investigated via fluorescence microscopy and FACS analysis. Cells derived from three cloned puppies, which were visually identified as transgenic, showed strong EGFP expression, whereas the others showed no GFP expression (Fig 3C and 3D). Strong *in vivo* expression of EGFP was also detected by immunostaining of muscle and brain tissue from BTF-967 (Fig 3E). Taken together, all positive transgenic clones, which had EGFP regulated by CMV promoter expressed a detectable fluorescence signal.

### Transcriptional activity of CMV promoter was maintained in dogs, but not *in vitro* culture occasionally

**Fig 4:**
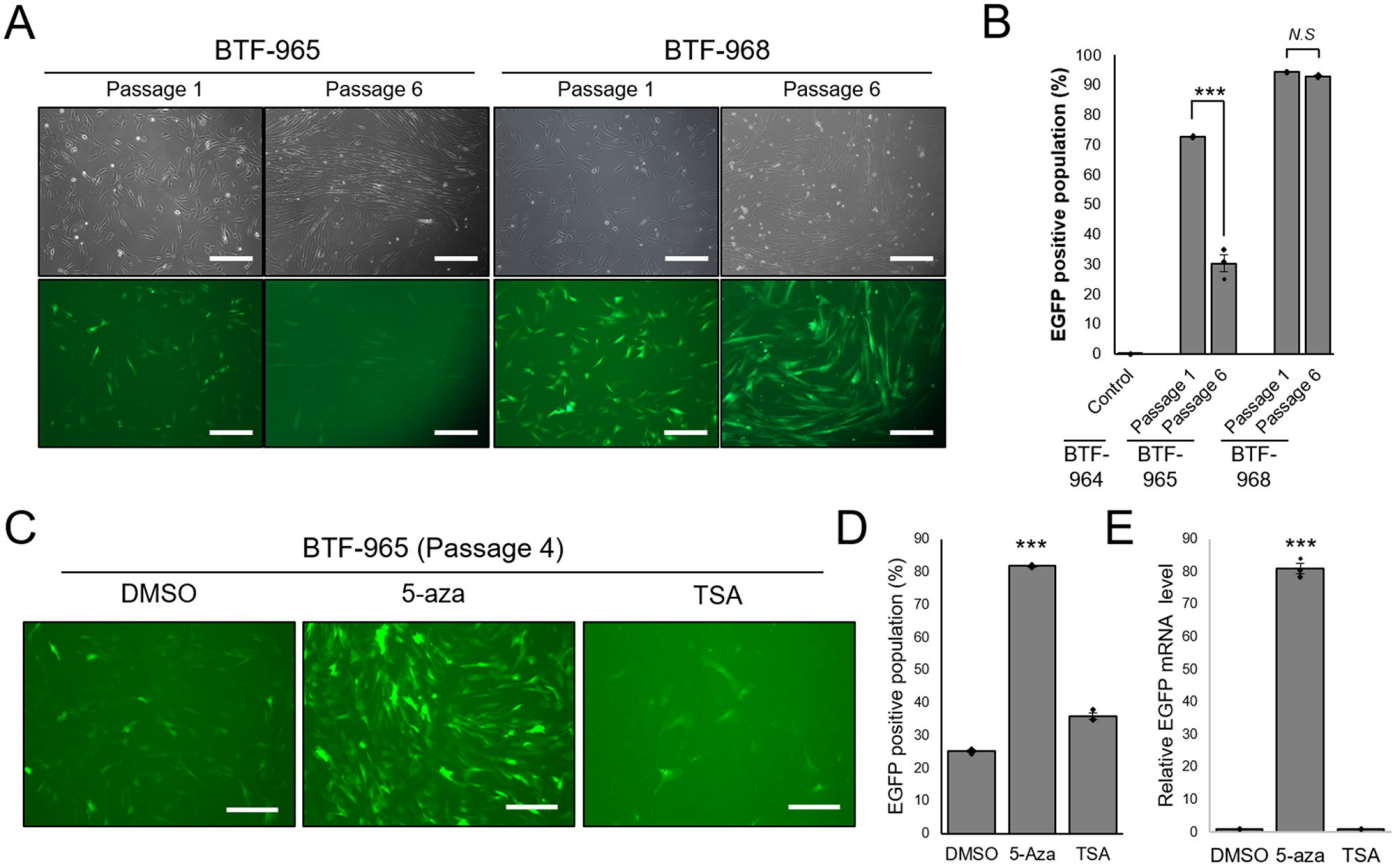
Occrurrence of DNA methylation mediated-CMV promoter silencing in fibroblasts isolated from BTF-965 during *in vitro* long term culture. (A) Image representing an attenuated EGFP signal in BTF-965 fibroblasts at passage 6, but not in BTF-968 fibroblasts. Scale bars indicate 50 μm. (B) FACS analysis verifiying EGFP positive population of each fibroblast line at indicated passages. BTF-964 fibroblasts are negative control. (C) Representative images of reactivated EGFP expression and (D) FACS analysis of rescued EGFP positive population in BTF-965 fibroblasts at passage 5 after treatment of 10 μM 5-Aza or 0.2 μM TSA for 72 h. Scale bars indicate 50 μm. (E) Quantitative PCR anlaysis to detect EGFP mRNA levels using RNA samples from each indicated cell line. *N*.*S*. indicates no significance. ***, *P* < 0.001.

A previous report showed that CMV promoter sequence is prone to be silenced by DNA methylation in transgenic pig [14]. So, we had consistently observed EGFP expression of the isolated fibroblast *in vitro*. Interestingly, fibroblast cells isolated from BTF-965 gradually lost fluorescence signal during *in vitro* culture, whereas cells from BTF-968 maintained strong EGFP expression constantly (Fig 4A and 4B). To test whether gradual silencing was caused by epigenetic modification, fibroblasts isolated from BTF-965 were treated with 5-Aza and TSA. The results showed that inhibitor of DNA methyltransferase rescued and strongly enhanced EGFP expression of transgenic fibroblasts (Fig 4C and 4D). Results of quantitative PCR indicated expression of EGFP gene was increased by transcriptional reactivation (Fig 4E). By performing inverse PCR, CMV-EGFP transgene integration sites in BTF-965 and BTF968 dogs were identified on chromosome 18 and on chromosome 8, respectively (S4–S6 Appendices). Therefore, our results imply that CMV promoter-driven transgene expression during *in vitro* culture condition could vary depending on the transgene integration site and a DNA methylation status.

**Fig 5:**
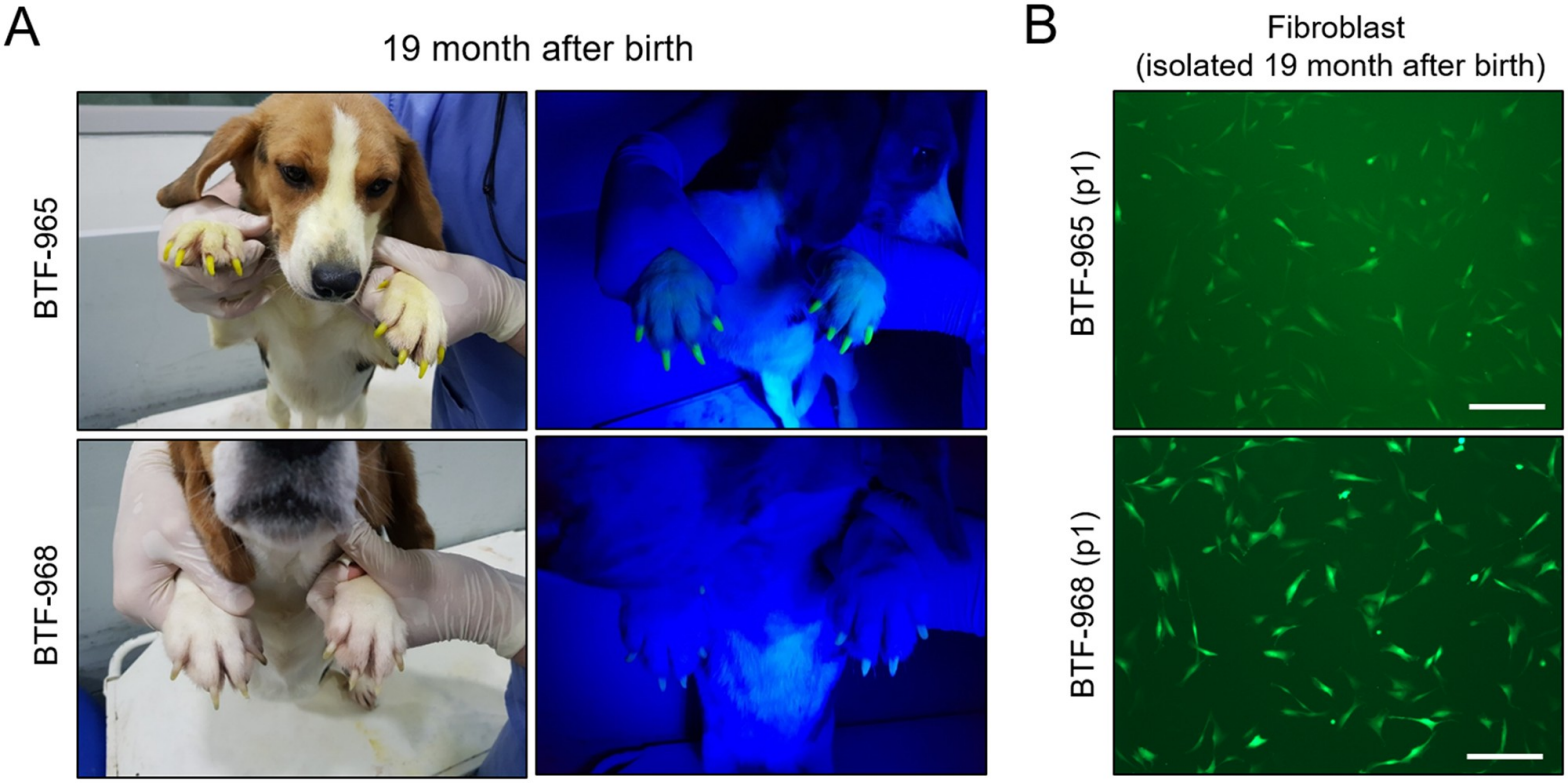
Persistent and stable expression of CMV promoter sequence-driven EGFP in individual SCNT transgenic adult dogs. (A) Images indicate detection of EGFP expression under ultraviolet light exposure in claws of BTF-965 and -968 at the age of 19 months. (B) Green fluorescence images of the newly isolated fibroblasts from BTF-965 and -968 dogs at 19 months. Scale bars indicate 50 μm.

Next, we verified if the transgene silencing appeared in grown-up BTF-965 and BTF-968 dogs. We could observe strong EGFP expression in their claws 19 months after birth under ultraviolet light (Fig 5A). Then, we also isolated fibroblasts at that period to confirm whether their fluorescence expression was detectable at the cellular level. Fluorescence images showed that both the newly isolated fibroblasts showed strong EGFP intensity, indicating stable transcriptional activity of CMV promoter sequence in transgenic dogs (Fig 5B). Taken together, it was evident that CMV promoter sequence stably and strongly regulated transgene expression in SCNT dogs without decreasing its transcriptional activity *in vivo*.

## Discussion

The stable expression of a transgene is crucial in transgenic animal models. However, many previous studies have shown that transgene expression is suppressed in a variety of transgenic organisms, including *C*. *elegans*, various mammals, and even plants [25–28]. Transgene expression has been seen to be incomplete or absent, even though vector constructs have been successfully integrated into host genomes. To overcome this obstacle in the generation of transgenic model, and to determine if commonly used promoters could be efficient in canine models, we compared the hEF1α and CMV promoter sequence by detecting EGFP transgene in SCNT-mediated transgenic dogs. Due to the short life span of canine fibroblasts, conducting SCNT using a heterogeneous donor cell population right after the transfection was the only option to produce transgenic dogs [29, 30]. Our results clearly showed that the CMV promoter strongly expressed EGFP transgene in all transgenic organisms, but the hEF1 promoter was completely silenced due to a DNA methylation.

Xia *et al*. suggested that the expression of a transgene, regulated by various promoter sequences in a lentiviral vector, was suppressed in human embryonic stem cells (ESCs) after integration into the host genome, and that its decrease was a highly promoter-dependent phenomenon [31]. The hEF1α promoter that we used in this study has been described as one of the most effective promoters for mammalian transgenic models [32]. In particular, transgene expression regulated by the hEF1α promoter has been reported to be highly stable and robust during differentiation of human and mouse ESCs [33, 34]. The hEF1α promoter also resulted in higher transgene expression in transgenic pigs, which was explained to be due to a hypomethylated status [35]. On the contrary, EGFP transgene expression governed by the CMV promoter, not by the EF-1α promoter, was strongly detectable in offspring generated from gene-modified male mice via lentivirus-mediated male germline stem cell manipulation [36]. Thus, there is a need to confirm which promoter sequence is the most suitable for transgenic dog. Because it has been known that the hEF1α promoter was more strong and stable in transgenic large animals produced by SCNT, we initially constructed a transgene expression cassette driven by the hEF1α promoter sequence for gene expression in transgenic dogs [35]. Unexpectedly, unlike previous reports, our results indicated reduced transcriptional activity of the hEF1α promoter, to the point that there was no detection of EGFP in SCNT transgenic dogs and their fibroblasts. Transgene expression is silenced by various mechanisms including DNA methylation, histone modification, position-effect variegation, and even transcriptional repression [13, 37]. As EGFP expression was rescued by DNA methyltransferase inhibitor, our results showed that the hEF1α promoter was likely silenced by methylation of the hEF1α promoter sequence.

Although the CMV promoter has been widely used in transgenic cell lines, due to its strong transcriptional activity, many studies have shown that it has a tendency to lose its transcriptional activity in transgenic cell lines and animals and this is theorized to be due to gradual methylation at its CpG sites [14, 38–40]. Our findings, contradictory to the literature, illustrated a considerable CMV promoter-mediated transgene expression in our transgenic dogs as well as in a previous canine models of type 2 diabetes [20]. It has been reported that the transgene expression levels and the methylation status of promoter are affected in a mouse strain-specific manner, even when using the same promoter sequence [41]. The murine model-based study implys that the promoter sequence for optimal transgene expression may vary depending on the genetic background of the host animals. Together, this difference in activities of each constitutive promoter depending on genotypes of host animals could explain the difference of CMV promoter activity between canine and other animal models, indicating there may be more factors involved than genetic predisposition to methylation or transgene silencing alone.

Interestingly, we observed a different tendency related to CMV promoter silencing activity during *in vitro* culture. This observation may be due to the random integration of the transgene into the host genome causing a ‘positional effect’, elucidating a gene expression variation from an interaction with its neighboring genetic contents [42]. To overcome this unpredictable variation, it would be better to develop efficient genetic engineering tools to knock-in into stable genomic loci called ‘safe harbor loci’ such as ROSA26 for dogs [43]. This site-specific gene insertion allows more stable transgene expression as well as prevention of a disruption of certain endogenous genes via a random transgene integration, which might cause development of unintended cancers or other genetic diseases as shown in BTF-967 [44].

In summary, the hEF1α and the CMV promoter sequences were tested in SCNT transgenic dogs. Our results indicated that the CMV promoter provides a more stable transgene expression in each transgenic dog. Therefore, we suggest that the CMV promoter is a more appropriate promoter sequence, than the hEF1 promoter, for the production of effective canine transgenic models via SCNT.

## Supporting information

S1 Appendix Full length PCR gels to verify the transgenic pups, shown in Fig 2B. Target products are indicated in red arrow.
(PDF)

S2 Appendix Full length PCR gels of semi-PCR samples using isolated mRNA from indicated cells to verify reactivation of the CMV promoter after treatment with 10 μM 5-Aza and/or 0.2 μM TSA for 72 hours, shown in Fig 2H. Target products are indicated in red arrow.
(PDF)

S3 Appendix Full length PCR gels to verify transgenic pups, shown in Fig 3B. Target products are indicated in red arrow.
(PDF)

S4 Appendix A sequencing result of inverse PCR product amplified using BTF-965 genomic DNA template. Genomic DNA sequeunce in the result is indicated in red color.
(PDF)

S5 Appendix A sequencing result of inverse PCR product amplified using BTF-968 genomic DNA template. Genomic DNA sequeunce in the result is indicated in red color.
(PDF)

S6 Appendix Summary of the sequencing results of inverse PCR. (PDF)
