# Supplementary figures and images for: Transcriptional activities of human elongation factor-1α and cytomegalovirus promoter in transgenic dogs generated by somatic cell nuclear transfer
Source: PLoS One. 2020 Jun 3;15(6):e0233784. doi: 10.1371/journal.pone.0233784 (PMC7269240; doi:10.1371/journal.pone.0233784)

# S1 Appendix

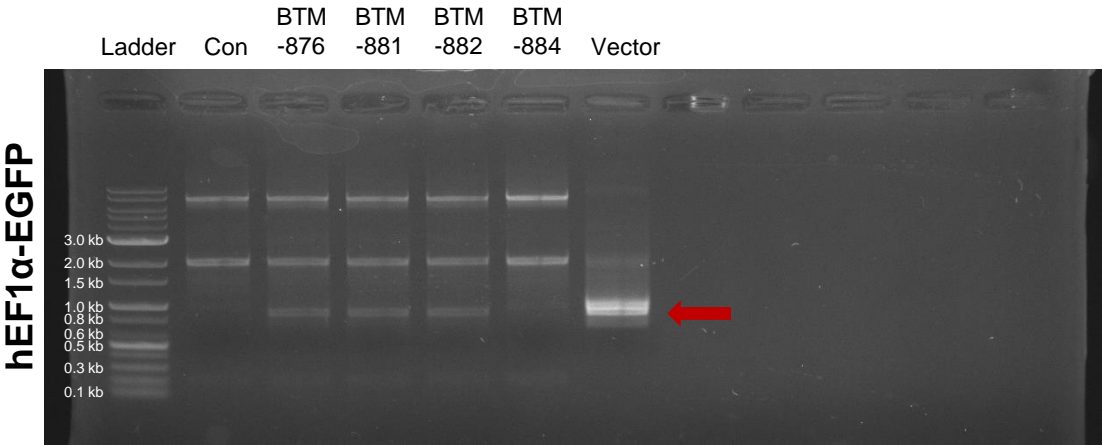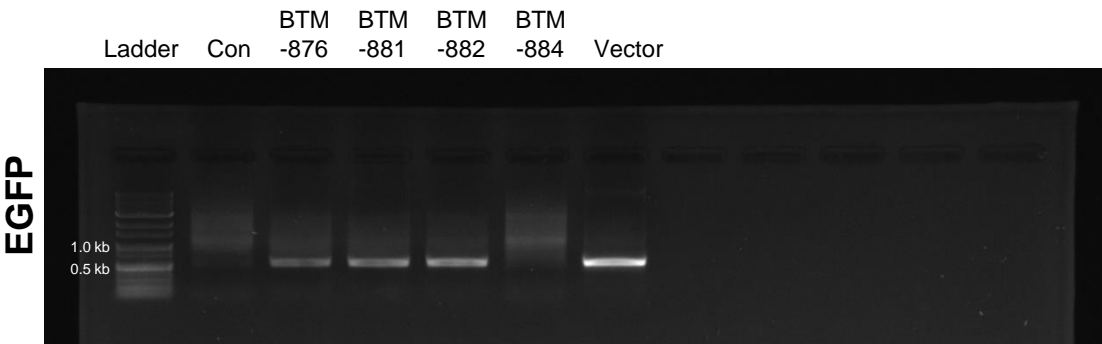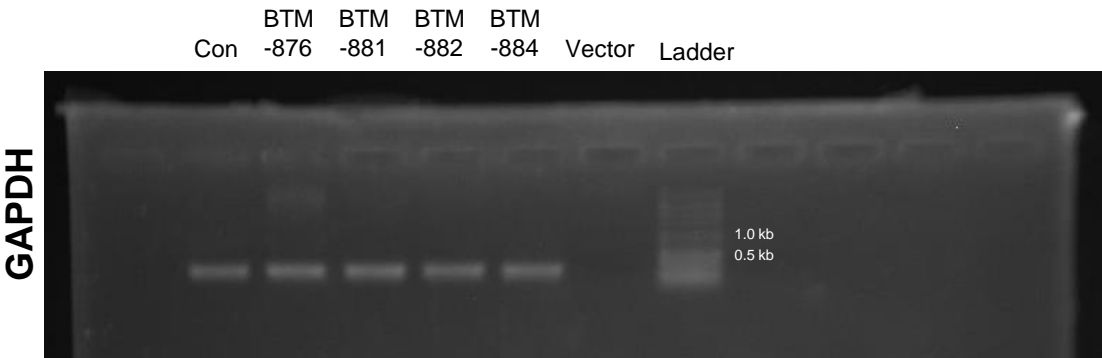

Supplement: S1 Appendix — Target products are indicated in red arrow. (PDF) [file pone.0233784.s001.pdf]

# S2 Appendix

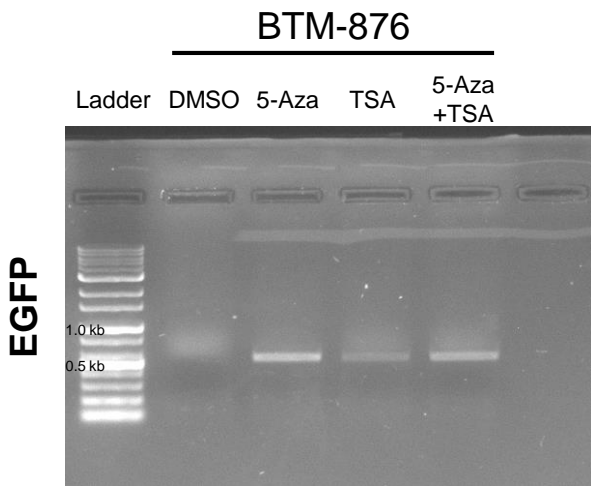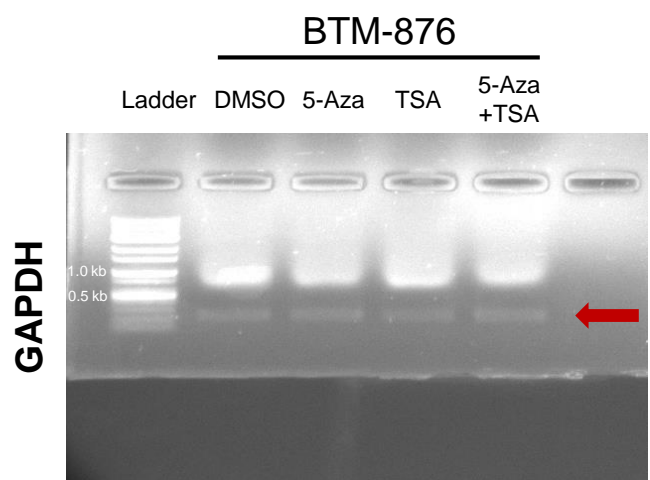

Supplement: S2 Appendix — Target products are indicated in red arrow. (PDF) [file pone.0233784.s002.pdf]

# S3 Appendix

CMV-EGFP

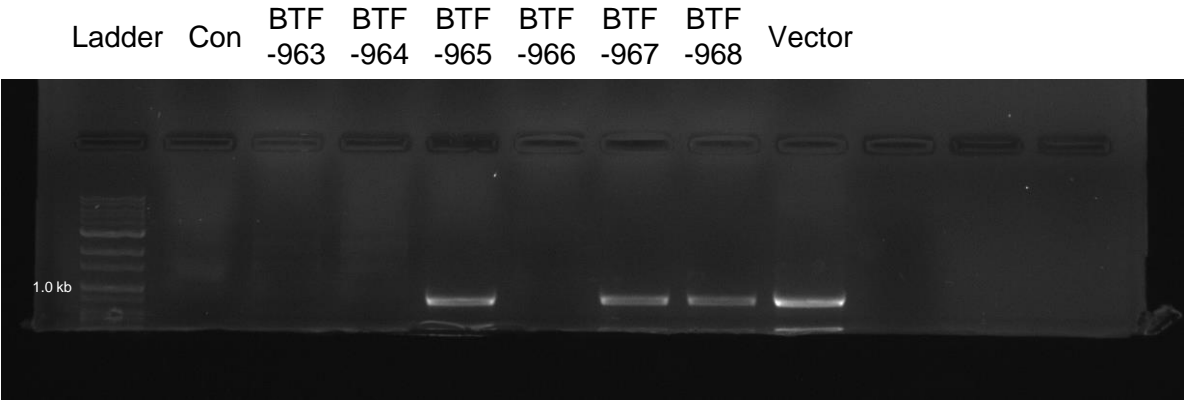

EGFP

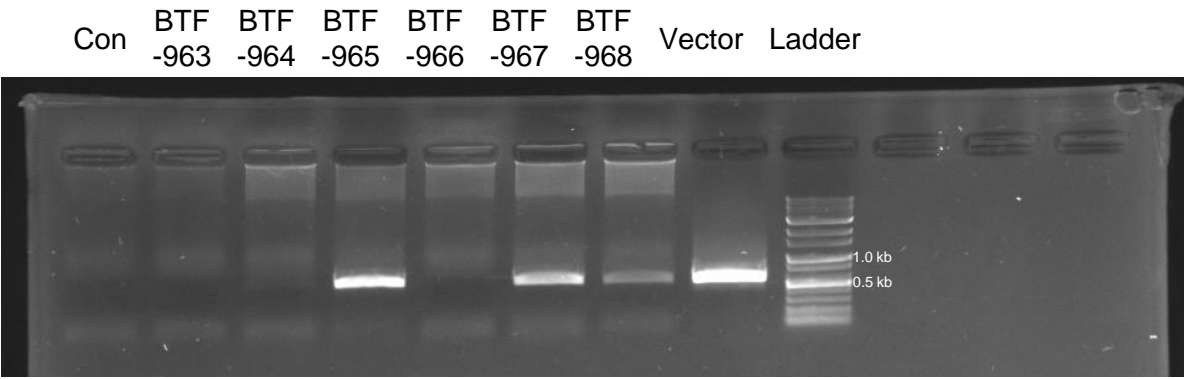

GAPDH

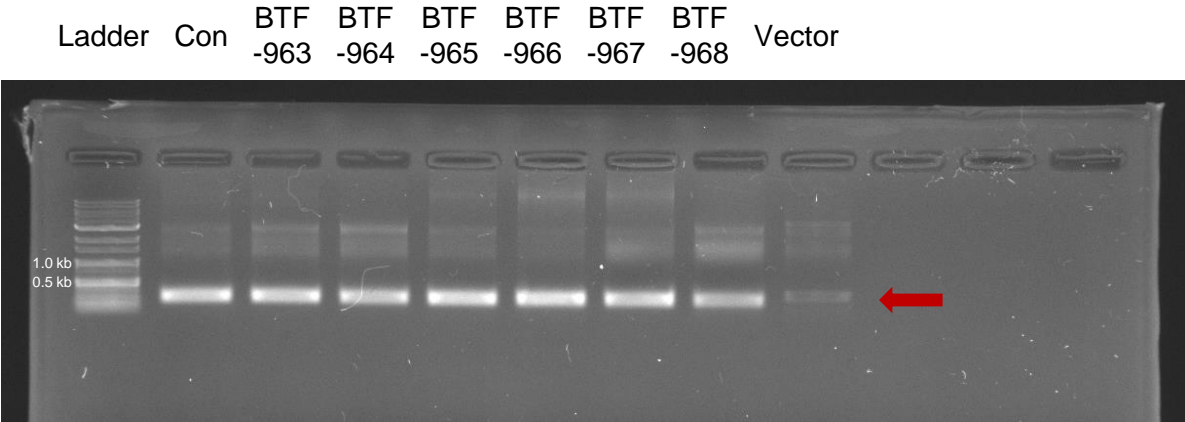

Supplement: S3 Appendix — Target products are indicated in red arrow. (PDF) [file pone.0233784.s003.pdf]

# S4 Appendix

- Inverse PCR result(BTF-965)

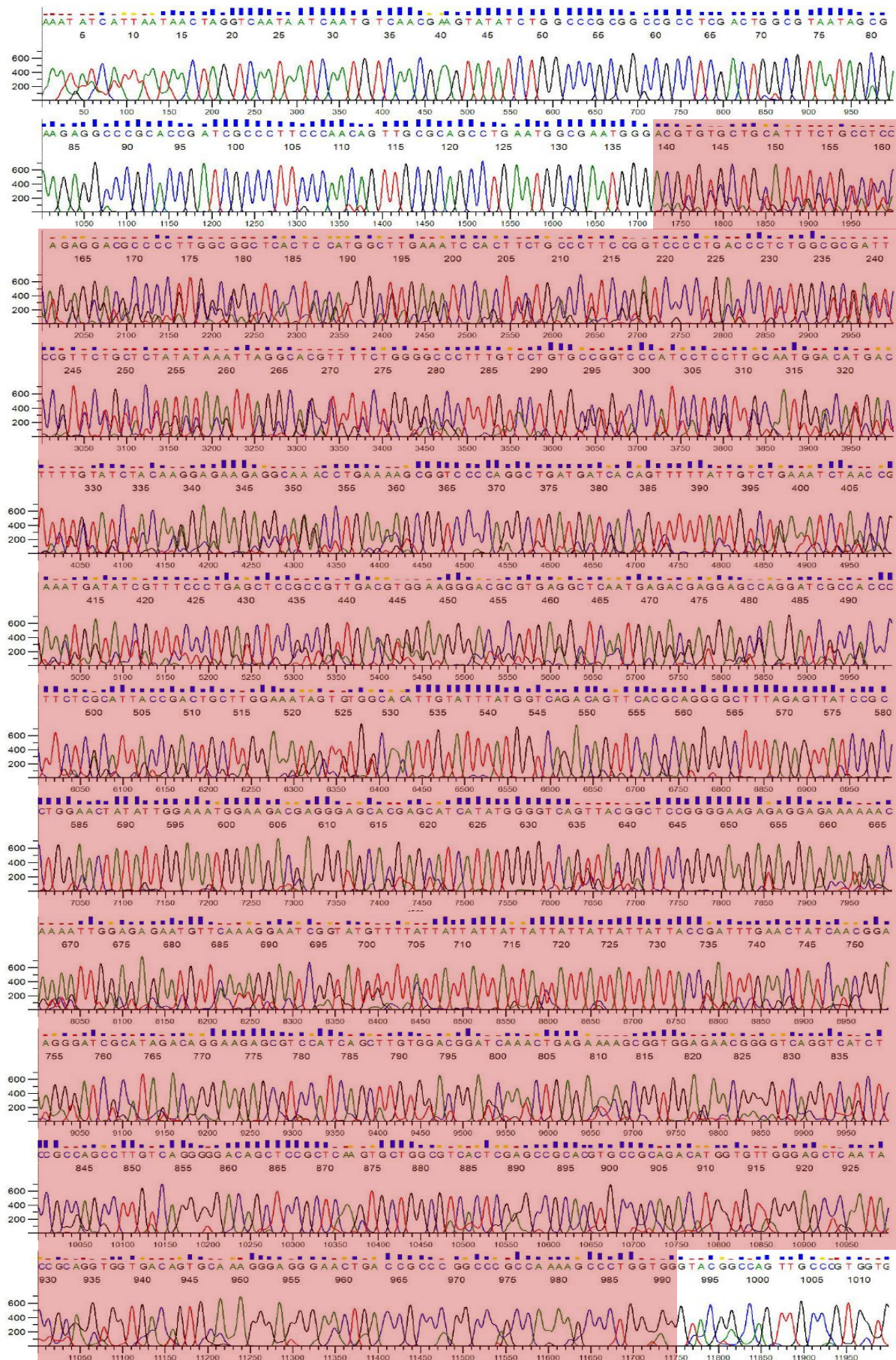

Supplement: S4 Appendix — Genomic DNA sequeunce in the result is indicated in red color. (PDF) [file pone.0233784.s004.pdf]

# S5 Appendix

- Inverse PCR result(BTF-968)

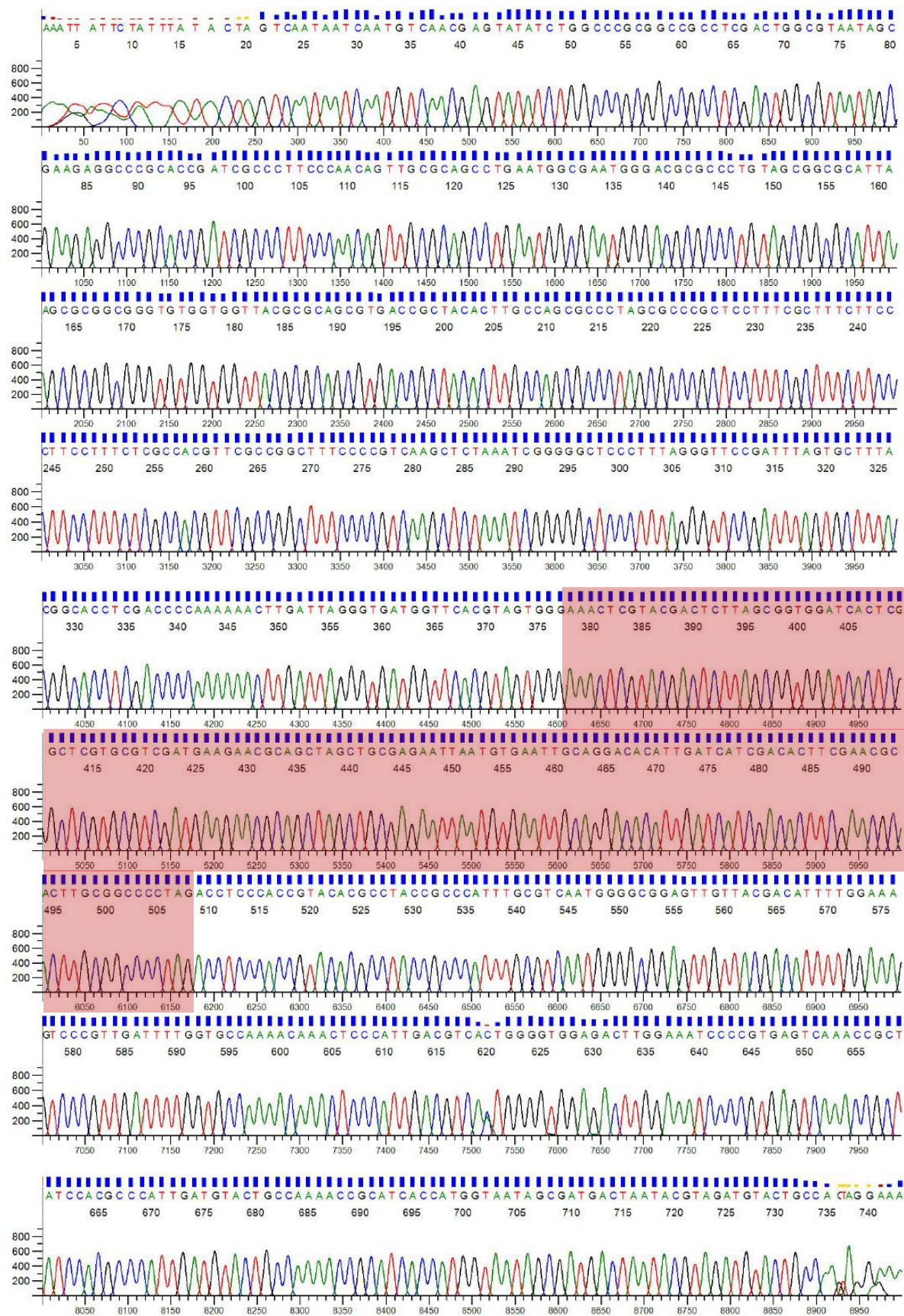

Supplement: S5 Appendix — Genomic DNA sequeunce in the result is indicated in red color. (PDF) [file pone.0233784.s005.pdf]
