# Supplementary material for: Transcriptional activities of human elongation factor-1α and cytomegalovirus promoter in transgenic dogs generated by somatic cell nuclear transfer
Source: PLoS One. 2020 Jun 3;15(6):e0233784. doi: 10.1371/journal.pone.0233784 (PMC7269240; doi:10.1371/journal.pone.0233784)
Supplement: S6 Appendix — (PDF) [file pone.0233784.s006.pdf]

S6 Appendix

- ## Inverse PCR result(BTF-965)

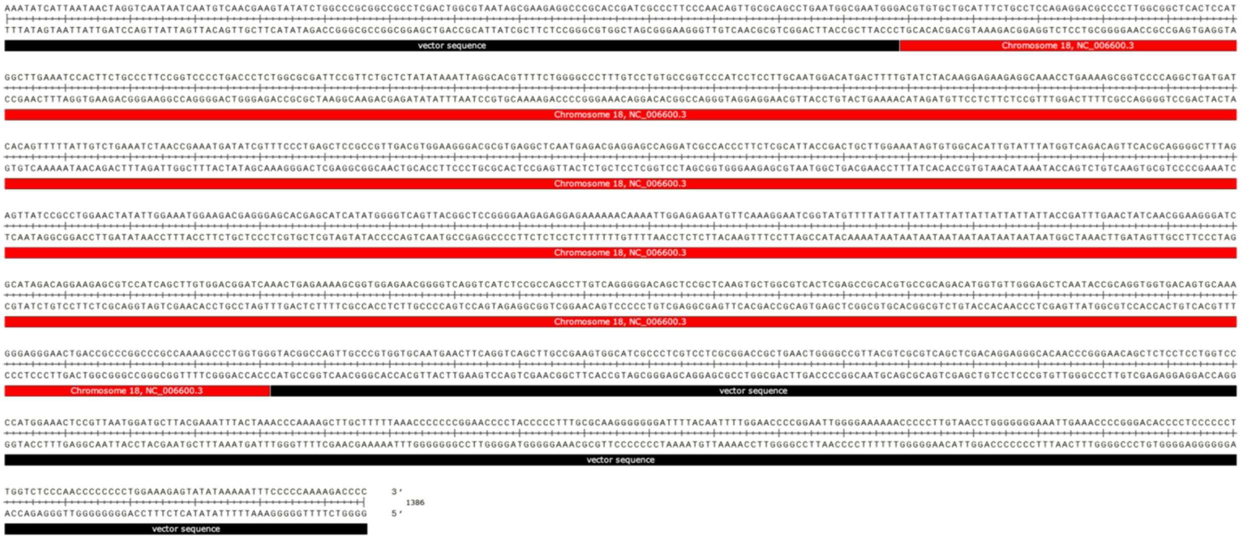

➢ **Black** sequence : transgene
➢ **Red** Sequence: canine genomic sequence
Chromosome 18
NCBI Reference Sequence: NC_006600.3
Range: 1566741 to 1567601 (93% match)
105042 bp at 5' side: zona pellucida-binding protein 1
26331 bp at 3' side: uncharacterized protein C7orf72 homolog isoform X4

- ## Inverse PCR result(BTF-968)

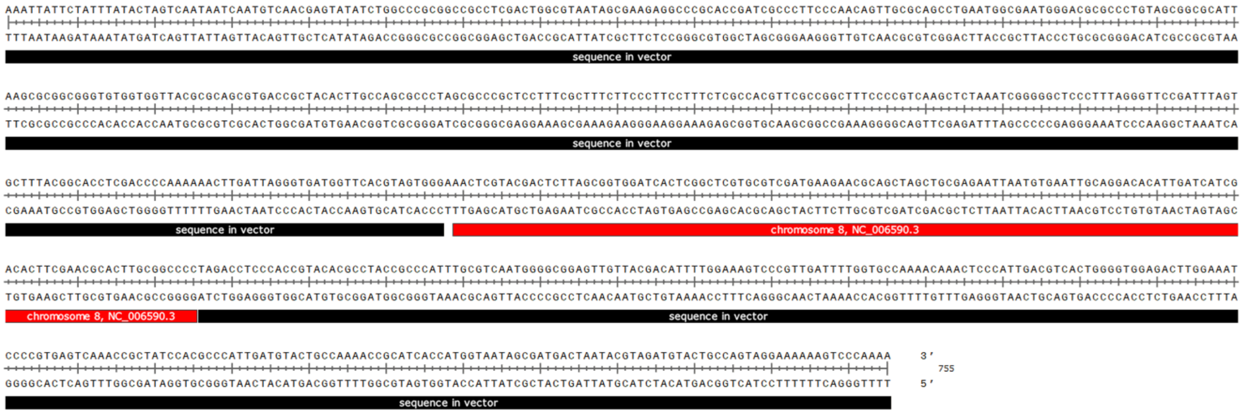

➢ **Black** sequence : transgene
➢ **Red** Sequence: canine genomic sequence
Chromosome 8
NCBI Reference Sequence: NC_006590.3
Range: 894450 to 894571 (85% match)
97616 bp at 5' side: olfactory receptor 4L1-like
16259 bp at 3' side: cation channel sperm-associated protein subunit beta
